# Hospital Contracts and Prices

**Published:** 1910-07-02

**Authors:** 


### HOSPITAL CONTRACTS AND PRICES.

### SURGICAL DRESSINGS.

THE correspondent referred to in our issue of June 18 who inquired if we could furnish the names of the firms who supplied surgical dressings at the prices quoted in our article of May 7 has, in response to our request that dressings and quantities should be specified, now written as follows:—

In answer to your letter of last week, I beg to inform you that the dressings I wish to inquire about are: white gauze, white gauze for packing, and gutta-percha tissue. We use from 2,000 to 2,500 yards of white gauze in the month and from 1 doz. to 2 doz. 12-yard packets of plugging gauze, and 1½ lb. of gutta-percha tissue.

A second correspondent writes as follows:—

With reference to the letters on the prices of surgical dressings, I would be most grateful if I might have the name of some firms which you recommend. We use white absorbent wool, taking 1 cwt. at a time, white gauze 240 yards, white lint 24 lb. Also can you advise as to the purchase of really reliable and not expensive mackintosh sheeting? I have found your articles most helpful and interesting.

We are very glad to comply with the requests of our correspondents, and we have sent them by post the names of firms from whom they can obtain samples of the dressings referred to in our article, and quotations. The quantities purchased by both correspondents are comparatively small, and there is just a possibility that they may, in consequence, have to pay a trifle more than the prices quoted in the article. It will depend, of course, in great measure upon the cost of carriage, the prices quoted including free delivery. We recommend our correspondents to try to increase the quantity they order at one time if they have storage accommodation, as more favourable terms can usually be obtained if a large order is placed. They might perhaps find it an advantage to make a six months' contract, undertaking to accept the whole in two or, at the outside, three deliveries. The terms of payment should be cash in one month.

We shall be glad if our correspondents will inform us of the result of their inquiries.

We find a little difficulty in replying to the question respecting a reliable and not expensive mackintosh sheeting. The rubber market for many years has had a strong upward tendency, and during the last few months prices have gone up by leaps and bounds, consequently we hesitate to recommend as reliable any cheap mackintosh sheeting. Our belief is that for the hard wear of a hospital ward the best procurable is the cheapest in the long run. Several of our large hospitals have for some years been using an expensive red-rubber sheeting, the administrators of those hospitals being of opinion that, in spite of the high price, money has been saved by so doing. Whether a red or a white mackintosh is used, we recommend our correspondent to avoid the cheap qualities, as we feel sure they will not prove economical. We have supplied our correspondent with the names of two or three reliable firms who would furnish quotations for rubber goods.

[We invite questions relating to any matter coming within the scope of this department.—ED., THE HOSPITAL.]

